# Genetic and Phenotypic Selection Affect Natural (Auto-) Antibody Reactivity of Chickens

**DOI:** 10.1371/journal.pone.0072276

**Published:** 2013-09-11

**Authors:** Britt G. de Jong, Aart Lammers, Leonora A. A. Oberendorf, Mike G. B. Nieuwland, Huub F. J. Savelkoul, Henk K. Parmentier

**Affiliations:** 1 Adaptation Physiology Group, Department of Animal Sciences, Wageningen University, Wageningen, The Netherlands; 2 Cell Biology and Immunology Group, Department of Animal Sciences, Wageningen University, Wageningen, The Netherlands; Auburn University, United States of America

## Abstract

Specificity, antibody isotype distribution and levels of natural antibodies (NAb) may be potential informative parameters for immune mediated natural disease resistance, immune modulation, and maintenance of physiological homeostasis. A large proportion of mammalian NAb have affinity for or are directed against self-antigens; so called natural auto antibodies (N(A)Ab). In the present study we showed the presence and typed levels and isotypes (total immunoglobulins, IgG and IgM) of N(A)Ab in plasma binding the ‘auto-antigen’ complex chicken liver cell lysate (CLL) of one-year old chickens from different genotype and phenotype backgrounds by ELISA and quantitative Western blotting. Higher levels of N(A)Ab binding CLL were found in plasma from chickens genetically selected for high specific antibody responses. In all birds, extensive staining patterns of plasma antibodies binding CLL were found for all isotypes, with IgG binding the highest number of CLL antigens and also showing the highest variation in staining patterns between individuals. Patterns of IgM antibodies binding CLL appeared to be more similar in all lines. Significant differences of binding patterns of N(A)Ab (antigen fragments of CLL and staining intensity) were detected between the different chicken lines, and lines could be clustered on the basis of their auto-antibody profile. In addition, also individual differences within lines were found. The present results indicate that analysis of the levels and the N(A)Ab repertoire of poultry like in mammals could provide a new way of distinguishing differences of immune competence and immune maturation between individuals, and could provide tools to select birds for health traits, or optimize hygiene and husbandry procedures.

## Introduction

Natural antibodies (NAb) are defined as antibodies present in normal healthy animals under the absence of (previous) deliberate antigenic stimulation or infection [1], [2]. Levels and isotypes of NAb in fish [3], cattle [4] and poultry [5] were suggested to be potential informative immune parameters for natural disease resistance. Because of their capacity to bind a wide range of evolutionary conserved but not chemically related molecules, NAb were regarded as (specific) part of the innate immune system, which provides effective and broad protection without previous exposure to a pathogen [2], [6], [7], [8]. In ‘lower’ vertebrates, NAb are mostly of the IgM isotype, while in higher vertebrates also IgA and IgG NAb were reported [7], [9]. The secretion of IgM may be induced independently of external antigenic stimulation, while IgG and IgA NAb secretion may be related with antigen stimulation [2].

A large proportion of mammalian NAb have affinity for or are directed against self antigens [10]. These so-called natural auto antibodies (N(A)Ab) may inactivate cytokines, mask auto-antigens, and clear obsolete or damaged cells and metabolic waste as part of anti-tumour surveillance or maintenance of homeostasis [7], [10]. NA(A)b are always present in the body albeit lower levels are found in young individuals, but levels may increase with age. In man and mice, quantitative (Western) immunoblotting has been used to analyse the N(A)Ab repertoire of individuals and inbred strains to various tissues like liver, kidney, brain and muscle [11], [12], [13], [14]. In general, the binding repertoire and levels of IgM binding auto-antigens increased during aging and remained stable later on, resulting in corresponding repertoires between individuals [15]. IgM patterns appeared to evolve without exogenous stimulation, confirming the notion that they are not formed randomly [16]. Auto-IgG patterns contained the same bands as IgM with the addition of more specific bands. Such IgG patterns were not expanding with age and remained stable at a young age [15]. This suggested that auto-IgG profiles could represent an ‘antibody fingerprinting’ of each individual [17]. Mice from different genetic strains [18] showed different auto-antibody binding profiles suggesting a genetic component underlying the N(A)Ab repertoire.

Earlier we reported auto-reactivity to various tissues in chicken plasma [19]. Chicken natural auto-antibodies were prone to *in vivo* and *in vitro* post-translational polymorphism [20], i.e. binding specificity of total immunoglobulins in plasma to various antigen fragments of the auto-antigen chicken liver cell lysate (CLL) changed after *in vitro* maltreatment (low pH or presence of H_2_O_2_) or *in vivo* challenge of birds with inflammation-inducing agents. In the present study we determined the presence and levels of total immunoglobulins (IgTotal), and the antibody isotypes IgM and IgG in plasma of approximately one-year old chickens from two lines that were divergently selected for high (Hg line) or low (Lg line) specific antibody agglutination titres to sheep red blood cells (SRBC) at 5 days after subcutaneous immunization with SRBC at 5 weeks of age, next to a random bred control (Cg line). These lines differ in almost every aspect of immune responsiveness. Earlier we found higher NAb and N(A)Ab levels at all ages in the Hg line as compared to the Cg and Lg lines [19]. In addition, we selected C line birds for (phenotypic) high (Ch) or low (Cl) plasma natural antibody levels to the model antigen keyhole limpet hemocyanin (KLH) at 16 weeks of age. The purpose of our study was threefold, first we tested line differences in levels and binding profiles of total plasma antibodies (IgTotal) and the isotypes IgM and IgG to the auto-antigen CLL, second we correlated the staining patterns of IgM and IgG antibodies, and third we clustered binding profiles of the antibody isotypes: IgTotal, IgM and IgG per line using principal component analysis. Our data suggest that groups (in the current study genotypes) or individual birds can be characterized by auto-antibody profiles. This offers opportunities to relate auto-antibody profiles with genotypes, and phenotypic traits such as immune responses, disease resistance, and (metabolic) disorders in chickens.

## Materials and Methods

### Plasma samples

Plasma of 45 (ELISA), and 58 (Western blotting) approximately one-year-old laying hens was used. Birds were kept under normal housing conditions with free roaming. The hens were from three different lines, which were either divergently bred during 29 generations for high (H line) or low (L line) primary (agglutinating) antibody responses at day 5 after primary intramuscular immunization with SRBC at 37 days of age, or a random bred control (C line). These represented genotypic high (Hg), genotypic low (Lg) hens, and genotypic control (Cg) hens. Average anti-SRBC agglutination titres were 13, 6, and 0 for the Hg, Cg, and Lg lines, respectively. In addition, from the C line population, birds were selected with high or low natural antibody levels at 16 weeks of age to the antigen keyhole limpet hemocyanin (KLH): a phenotypic high control line (Ch, with an average ELISA based titre of 5.34) and a phenotypic low control line (Cl with an average ELISA based titer of 0.93) (data not published). Plasma samples, frozen liver tissue and frozen brain tissue from a five week old hen resided from an experiment approved by the Animal Welfare Committee of Wageningen University: DEC approval 2011022b.

### Reagents

Chicken liver cell lysate (CLL) and chicken brain lysate were made by freezing 0.5 g liver tissue from a five week old C line hen in liquid nitrogen. The liver was lysed with 5 ml lysis buffer (50 mM Tris, 100 mM NaCl, 1 mM EDTA, 1 mM EGTA, 0.1% SDS, 2% Glycerol, pH 7.4) with addition of 2.5 ml protease inhibitor cocktail (Sigma-Aldrich, St. Louis, USA). The mixtures were centrifuged for 15 min (13,500 g) after which the supernatant containing CLL was taken of and kept at −20°C until use.

### Plasma levels of antibody isotypes binding chicken liver lysate (CLL)

Levels of total antibodies (IgTotal) and antibody isotypes IgM and IgG binding CLL in plasma of 15 hens of the Hg, Cg and Lg lines, respectively, were determined by an indirect enzyme linked immunosorbent assay (ELISA). Briefly, 96-well plates were coated with either 2, 4 or 8 µg/ml CLL. After subsequent washing with H_2_O containing 0.05% Tween, the plates were incubated for 60 min at room temperature with serial 2-step dilutions of plasma in PBS containing 0.5% horse serum (HS) and 0.05% Tween starting at a 1∶40 dilution of plasma. Binding of IgM, IgG and IgTotal antibodies to CLL was detected after 1 h of incubation at room temperature with 1∶25,000 in PBS (containing 0.5% HS and 0.05% Tween) diluted goat anti-chicken IgM coupled to horse radish peroxidase (PO) (GACh/IgM/PO) directed to the µ heavy chain of IgM (Bethyl, Montgomery, TX), or 1∶50,000 diluted goat anti-chicken IgG_Fc_ coupled to PO (Bethyl), or 1∶25,000 diluted rabbit anti-chicken IgG_H+L_ coupled to PO (RACh/IgG_H+L_/PO (Bethyl), respectively. After washing, tetramethylbenzidine and 0.05% H_2_O_2_ were added and incubated for 10 min at room temperature. The reaction was stopped with 50 µL of 1 M H_2_SO_4_. Extinctions were measured with a Multiscan G0 instrument (ThermoFisher Scientific, Vantaa, Finland) at a wavelength of 450 nm. A pooled known positive sample for CLL was used as positive standard and was included in each plate. Titers were expressed as log_2_ values of the dilutions that gave an extinction closest to 50% of E_max_, where E_max_ represents the highest mean extinction of a standard positive (pooled) plasma present on every microtiter plate.

### Natural (auto-) antibody profiles by Western blotting

Western blot analysis was used for the determination of natural (auto-) antibody binding profiles to CLL in plasma of 58 birds prior to immunization with rabbit gamma globulin (RGG). First, CLL was diluted 1∶30 and incubated 1∶1 v/v with sample buffer (1∶20 β-mercapthoethanol and Laemmli sample buffer (BIORAD, Hercules, USA)) at 95°C for 5 min. One aliquot was made and used for all the experiments to ensure the same quality of the sample. The CLL was separated on molecular weight in a Miniprotean Tetra cell (BIORAD) with 12.5% SDS-PAGE (sodium dodecyl sulphate-polyacrylamid gel electrophoresis) under reducing (β-mercapthoethanol) conditions. Molecular weight markers consisted of Precision Plus Dual Color Protein™ Standards (10, 15, 20, 25, 37, 50, 75, 100, 150, 250 kDa (BIORAD)).

After separation on molecular weight, fragments were blotted to a polyvinylidene fluoride membrane (PVDF, BIORAD) with a semi-dry transfer gel blotting system (Trans-blot SD, BIORAD) for 30 min at 15 V for 2 blots simultaneously. Blots were incubated overnight at 4°C in blotting buffer (PBS, 5% normal horse serum, and 0.05% Tween), or for 1 h on the rocking table at room temperature.

Blots were incubated with plasma samples in a 16 lane miniblotter (Immunetics, Boston, MA). Each lane was filled with 200 µL 1∶40 diluted plasma or dilution fluid (PBS, 0.5% normal horse serum and 0.05% Tween) to fill up empty lanes (negative control). As a positive control, one lane of normal rooster plasma was added. The miniblotter rotated for 1 h at room temperature. Thereafter, the blots were rinsed with dilution fluid five times for 10 minutes on the suspension mixer. Next, the second antibody (conjugate) was added to the blots and incubated for 1 h on the suspension mixer. The following conjugates to detect N(A)Ab isotypes were used; IgTotal (1∶500 diluted rabbit anti-chicken IgG_H+L_ coupled to horse radish peroxidase (PO), IgM: 1∶500 diluted rabbit anti-chicken IgM coupled to PO, and IgG: 1∶500 diluted rabbit anti-chicken IgG_fc_ coupled to PO all from Nordic, Tilburg, The Netherlands). After washing, bands were visualized with the Opti-4CN substrate kit (BIORAD) according to manufacturer's instructions.

### Analyses and Statistics

Plasma levels of antibody isotypes (IgTotal, IgM and IgG) binding CLL determined by ELISA were analysed by one-way ANOVA for the effect of line. Mean differences between lines were tested with Bonferroni's test. To analyse the number of bands and the staining intensities in Western blots, CLL blots were scanned with a flatbed scanner and saved as a tif file. The TotalLab v2006 software program (NonLinear Dynamics) was used, with a minimum slope of 75 and a linear log curve for identification of stained fragments, measurement of pixel intensities, calculations and graphs. Bands between 10 and 100 kDa were considered reliable, and fragments outside this range were not analysed. To detect significant differences of the extinction values between the binding profiles of the plasma antibodies from the five chicken lines to CLL, a one-way ANOVA was performed on the extinction values for the effect of line. Pearson's correlation adjusted for line was done to test correlations of extinctions per band between the three antibody isotypes. All statistical analyses were done with SAS 9.2 (proc GLM and proc Corr) [21]. In every blot, extinction values from the negative control lanes (dilution fluid) were subtracted from each fragment. The level of significance used was P<0.05.

Clustering of lines on binding profiles after Western blotting of the three antibody isotypes to CLL was done by principal component analysis (PCA) on log-transformed extinction data using the CANOCO package for Windows [22].

## Results

### ELISA

Table 1 shows least square means of the levels of plasma antibodies: total immunoglobulins (IgTotal) and levels of the antibody isotypes IgM and IgG binding CLL that were found in the birds of the Hg, Cg, and Lg lines. Significantly higher levels of all antibodies, regardless of CLL coating concentrations were found in the Hg line as compared to the Lg line, whereas the Cg lines was usually in-between or similar to the Hg or Lg line.

**Table 1:** Least square means plus standard error (SE) of immunoglobulin M (IgM) and immunoglobulin G (IgG) titers and titers of total antibodies (IgTotal) binding chicken liver lysate (CLL).

		IgTotal			IgM			IgG	
Line^1^	2^2^	4	8	2	4	8	2	4	8
Hg	5.51^a^	5.10^a^	4.87^a^	6.33^a^	6.58^a^	6.89^a^	5.97^a^	5.85^a^	5.96^a^
Cg	3.71^b^	3.55^b^	3.63^b^	6.43^a^	6.22^a^	6.03^a^	3.98^b^	4.25^b^	3.65^b^
Lg	2.08^c^	2.16^c^	2.17^c^	4.27^b^	4.07^b^	3.60^b^	3.31^b^	3.09^c^	2.84^b^
SE	0.39	0.28	0.24	0.34	0.32	0.31	0.39	0.37	0.33
Line^3^	***	***	***	***	***	***	***	***	***

a,b,c Lines differ significantly (*P*<0.05) when different superscript.

1 Hg = genotypic high hens, Cg = control hens, Lg = genotypic low hens, (N = 15 birds/line).

2 microgram/ml coating of CLL to ELISA plates.

3 Line effect:

*** P<0.001.

### Western blot profiles

**Figure 1:**
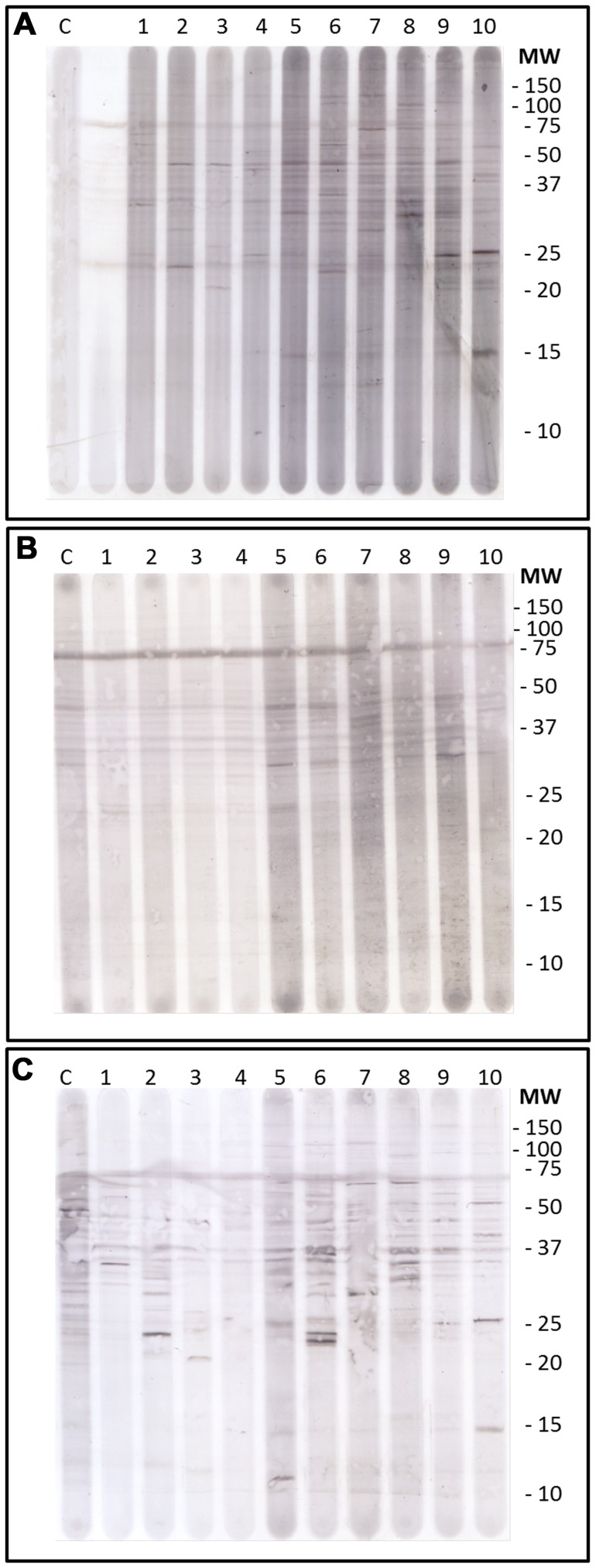
Western Blots representing the same set of birds for different isotypes. From top to bottom: A) IgTotal, B) IgM and C) IgG. Lanes 1–4: genotypically low (Lg) hens, 5–10: phenotypically high (Ch) hens, C = positive control (rooster plasma), m = marker, and other lanes are negative control (dilution fluid).

Western blot profiles were generated for the three isotypes IgTotal, IgM and IgG using plasma from birds of the five lines. In Fig. 1 representative blots are shown for each of these isotypes, where each similar numbered lane represents the same bird. Comparison of these three blots shows clearly that incubation of the blots with anti-IgG results in the most distinctive binding pattern, with many individual differences, also within lines. The IgTotal blots show some similarity with IgG, although less distinct. For IgM, there seemed to be more corresponding bands which are present in all chickens regardless of line. Also a difference in background between the first four animals (Lg line birds) and the last six (Ch line birds) is indicated.

**Figure 2:**
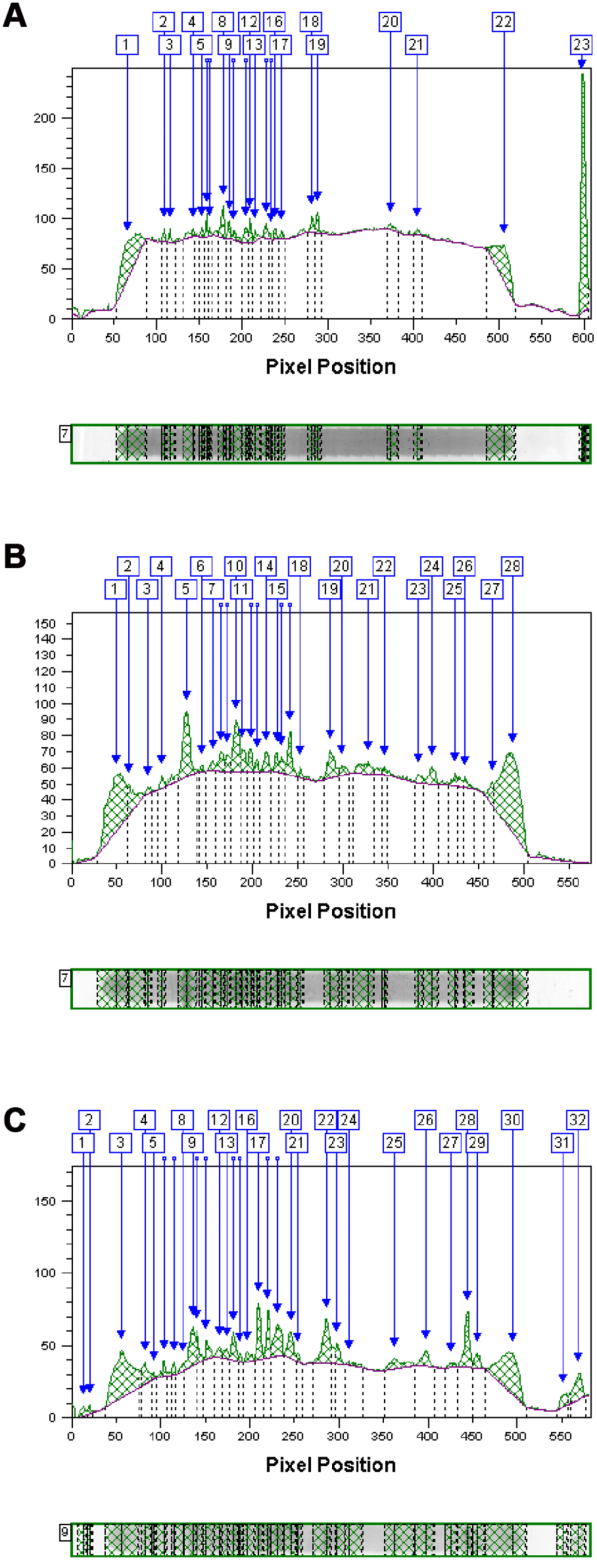
Graphic representation of Western Blots. On each figure the sample data from one phenotypically high (Ch) hen is shown for the three different isotypes. From top to bottom; IgTotal, IgM and IgG.

Each lane of these blots was analysed with the TotalLab software (NonLinear Dynamics), and representative graphs for each isotype are shown in Fig. 2. These lanes represent the binding pattern and extinction values of plasma antibodies to CLL from a phenotypic high C line hen. As expected, from Fig. 2, the same general findings can be deduced. IgG appears to show the highest number of stained fragments, followed by IgM and IgTotal. Fig. 2 also illustrates that although the baseline (background colouring) for IgG is lower than for IgM, the extinction intensities of the individual peaks from IgG were higher than for IgM.

The average number of bands per chicken differed between the different isotypes (Table 2). The largest number of stained fragments were found for the isotype IgG. This was in line with the general image that can be seen in the three blots and graphs described above (Figs. 1 and 2). For a general overview, Table 3 shows the average number of stained bands per chicken per line for the three isotypes. Genotypic low (Lg) hens showed on average the lowest number of stained bands. The genotypic (Hg) and phenotypically high (Ch) birds showed comparable numbers of stained fragments. Also genotypic (Lg) and phenotypic low (Cl) birds showed comparable numbers of stained fragments for isotypes IgM and IgG. The random bred control (Cg) line hens showed a comparable number of bands as genotypic low (Lg) hens for IgT. Numbers of bands bound by IgM and IgG in genotypic high (Hg) and phenotypic high (Ch) hens were alike.

**Table 2:** Average number of antibody recognized CLL bands per chicken, without correcting for the different lines.

Isotype	Average # bands per chicken
IgTotal	14.2
IgM	17.0
IgG	19.8

**Table 3:** Average number of bands per chicken, corrected for the different lines of chickens.

Isotype	Average # bands per chicken per line				
	Hg^1^	Cg	Lg	Ch	Cl
IgTotal	15.5	10.5	9	18.8	16.4
IgM	17.1	17.0	15.5	20.1	15.3
IgG	21.8	24	16.1	20.2	17.0

1 Hg = genotypic high hens (N = 12), Cg = control hens (N = 11), Lg = genotypic low hens (N = 11), Ch = phenotypic high hens (N = 12), Cl = phenotypic low hens (N = 12).

### Analyses of staining intensities of the profiles

Tables 4, 5 and 6 show the results of the significant differences between levels of binding intensity of antibodies in plasma of the five lines directed to CLL fragments. For each fragment, the average extinction per stained band by the chickens of each line is shown, followed by the standard error and the level of significance (P<0.05). Zero extinction implies the absence of extinction of that specific fragment relative to the background staining by all chickens of a particular line.

**Table 4:** Analysis of chicken plasma IgT binding CLL.

Band (kDa)	Line^1^						
	Hg	Cg	Lg	Ch	Cl	SE	P
93	0.00^b^	1348.18^b^	11759.36^a^	6230.83^ab^	0.00^b^	3065.74	*
88	0.00^b^	0.00^b^	0.00^b^	7984.92^ab^	15097.08^a^	3292.11	**
74	0.00^b^	0.00^b^	0.00^b^	7697.00^ab^	15692.17^a^	3591.63	**
62	9216.50^a^	1445.45^b^	2559.73^b^	1982.92^b^	0.00^b^	1980.53	*
57	5639.17^b^	11743.82^ab^	21862.91^a^	7487.25^b^	4416.25^b^	3526.56	**
44	9902.75^ab^	0.00^b^	0.00^b^	14276.83^a^	15904.83^a^	3803.63	**
43	9638.08^b^	18769.73^a^	0.00^c^	0.00^c^	0.00^c^	2130.17	***
39	8714.25^ab^	13087.55^ab^	1613.36^b^	19851.58^a^	19245.75^a^	4672.60	*
36	0.00^b^	4401.82^ab^	0.00^b^	11925.58^a^	4901.00^ab^	2785.15	*
34	30702.83^a^	17084.36^ab^	3287.18^b^	9727.25^b^	11831.08^ab^	5609.21	*
33	19008.33^ab^	0.00^c^	7531.91^bc^	29615.00^a^	8043.83^bc^	4218.89	***
32	0.00^b^	2940.36^b^	0.00^b^	2229.50^b^	19091.75^a^	3359.61	**
31	19294.00^a^	2178.27^b^	2553.09^b^	1277.83^b^	8263.83^ab^	4195.71	*
28	9666.58^b^	19432.82^ab^	35689.45^a^	5305.92^b^	1910.83^b^	5964.03	**
27	15883.50^ab^	0.00^b^	5245.00^b^	24951.58^a^	14186.17^ab^	5707.90	*
25.4	23669.75^a^	0.00^b^	0.00^b^	19875.83^a^	38060.08^a^	5482.90	***
23.5	3248.42^b^	0.00^b^	2222.18^b^	6974.25^b^	26795.58^a^	5296.32	**
23	9848.00^b^	4079.27^b^	11471.54^ab^	25288.00^a^	3001.75^b^	5279.99	*
22	6831.58^ab^	0.00^b^	0.00^b^	1735.75^b^	14181.58^a^	3643.55	*
21	5628.17^b^	0.00^b^	0.00^b^	22046.67^a^	16312.75^ab^	5748.26	*
20.5	0.00^b^	0.00^b^	0.00^b^	0.00^b^	6927.67^a^	1689.91	*
19.4	2131.08^b^	0.00^b^	1044.55^b^	9112.92^a^	0.00^b^	1863.64	**
17.7	12095.92^a^	0.00^b^	0.00^b^	0.00^b^	0.00^b^	2253.07	**
17	9830.92^ab^	0.00^b^	2476.82^b^	12245.83^ab^	19711.17^a^	4242.01	*
15.3	6661.83^b^	14740.91^b^	0.00^b^	9411.75^b^	43839.83^a^	6297.84	***
15	0.00^b^	0.00^b^	1851.91^b^	21534.75^a^	0.00^b^	4050.57	**
13	9823.83^b^	9659.91^b^	0.00^b^	3011.67^b^	33520.59^a^	3630.95	***
12.8	0.00^b^	0.00^b^	0.00^b^	9342.58^a^	11244.33^a^	2888.00	**
11.7	0.00^b^	3357.09^b^	0.00^b^	15753.08^a^	0.00^b^	3590.59	*
10	1888.00^b^	0.00^b^	0.00^b^	10320.83^ab^	16598.50^a^	3933.19	*

Per fragment (size kDa) the average extinction (least square means) per line of hens is shown, with standard error (SE) and P-value.

1 Line: Hg = genotypic high hens (N = 12), Cg = control hens (N = 11), Lg = genotypic low hens (N = 11), Ch = phenotypic high hens (N = 12), Cl = phenotypic low hens (N = 12).

Extinctions within a row with different superscripts^a,b,c^ differ significantly (P<0.05).

* P<0.05,

** P<0.01,

*** P<0.001.

**Table 5:** Analysis of chicken plasma IgM binding CLL.

Band (kDa)	Line^1^						
	Hg	Cg	Lg	Ch	Cl	SE	P
97	21906.45^a^	0.00^c^	2994.09^bc^	10432.67^b^	7995.92^bc^	3391.48	***
93	8582.27^a^	0.00^b^	0.00^b^	0.00^b^	0.00^b^	1869.09	**
76	1360.63^b^	2994.00^b^	0.00^b^	2000.08^b^	46184.67^a^	5023.82	***
66	46618.55^a^	9592.09^c^	19381.73^bc^	34449.58^ab^	15954.17^bc^	6564.17	**
62	9090.00^ab^	15107.09^a^	9382.27^ab^	2724.00^b^	2869.08^b^	3087.86	*
60	0.00^c^	1695.82^bc^	0.00^c^	10494.08^a^	9200.08^ab^	2999.82	*
53	14533.18^a^	4526.82^b^	14618.55^a^	6437.67^ab^	0.00^bc^	3224.24	**
50	28823.55^ab^	33791.73^a^	13441.09^c^	21561.50^b^	23712.75^abc^	4030.46	*
47	29071.55^a^	19130.73^b^	14860.91^b^	20086.67^ab^	15465.83^b^	3210.92	*
45	0.00^c^	12260.18^b^	8943.00^bc^	21146.42^ab^	26584.16^a^	3827.46	***
43	38670.00^a^	20582.82^b^	18151.82^b^	20069.25^b^	12204.08^b^	4115.95	**
40	26901.27^b^	39842.36^a^	23272.18^b^	24773.08^b^	19440.08^b^	3888.64	**
39	0.00^b^	0.00^b^	0.00^b^	0.00^b^	11131.42^a^	1985.45	***
35	17024.27^b^	10293.46^b^	7102.09^b^	12388.17^b^	32626.33^a^	4988.22	**
34	0.00^c^	0.00^c^	7037.55^c^	27820.42^b^	36685.92^a^	2679.01	***
33	50513.73^a^	39948.73^a^	16373.82^b^	9774.58^c^	24236.33^b^	4948.56	***
31	26611.72^a^	18499.73^ab^	15370.00^b^	17583.25^b^	5004.00^c^	2906.71	**
29	38831.45^a^	9276.27^b^	8109.64^b^	9378.92^b^	9564.42^b^	4582.97	***
27	20892.00^b^	39885.55^a^	1123.00^c^	9349.25^bc^	0.00^c^	6391.61	**
22	27958.36^a^	0.00^b^	20368.45^ab^	16229.67^ab^	8173.00^b^	6339.53	*
20	0.00^b^	0.00^b^	0.00^b^	5636.58^ab^	15755.08^a^	4137.91	*
17.7	0.00^b^	21133.45^a^	0.00^b^	4140.58^b^	4781.33^b^	4505.28	*
17.5	0.00^b^	3317.64^ab^	0.00^b^	9208.75^a^	0.00^b^	2372.92	*
15	4719.27^b^	31390.82^a^	5358.55^b^	21164.33^ab^	37350.33^a^	8146.52	*
12.3	34711.09^b^	64208.09^a^	0.00^c^	4713.00^c^	16456.50^bc^	7724.70	***
11.7	34012.00^a^	3870.45^b^	11163.64^b^	7694.42^b^	13792.42^b^	6250.33	*
11	3061.18^b^	1867.00^b^	5998.09^ab^	25644.83^a^	21302.42^a^	6225.12	*
10	11446.09^ab^	0.00^b^	0.00^b^	4099.00^b^	23820.92^a^	5177.81	*

Per fragment (size kDa) the average extinction (least square means) per line of hens is shown, with standard error (SE) and P-value.

1 Line: Hg = genotypic high hens (N = 12), Cg = control hens (N = 11), Lg = genotypic low hens (N = 11), Ch = phenotypic high hens (N = 12), Cl = phenotypic low hens (N = 12).

Extinctions within a row with different superscripts^a,b,c^ differ significantly (P<0.05).

* P<0.05,

** P<0.01,

*** P<0.001.

**Table 6:** Analysis of chicken plasma IgG binding CLL.

Band (kDa)	Line^1^						
	Hg	Cg	Lg	Ch	Cl	SE	P
97	12745.92^ab^	768.10^b^	1506.63^b^	4693.25^b^	20061.50^a^	3174.92	***
93	0.00^b^	6885.10^a^	618.10^b^	939.58^b^	0.00^b^	1538.72	*
88	2184.83^b^	1453.60^b^	3621.36^b^	2080.00^b^	12739.75^a^	2159.71	**
66	30574.92^a^	8280.20^b^	0.00^b^	7385.33^b^	23565.58^a^	4885.23	***
61	0.00^b^	0.00^b^	0.00^b^	1968.83^a^	0.00^b^	396.37	**
59	0.00^b^	5906.70^a^	5720.36^a^	4017.42^a^	0.00^b^	1550.69	*
47	5717.42^a^	2645.60^b^	1607.55^b^	6491.83^ab^	0.00^b^	1588.41	*
45	0.00^b^	3300.30^b^	13240.27^a^	12064.25^a^	13554.75^a^	2292.40	***
44	25056.92^a^	19276.40^a^	1545.91^b^	0.00^b^	5698.08^b^	3371.21	***
43	0.00^b^	0.00^b^	0.00^b^	4801.42^a^	3965.50^a^	1385.15	*
40	1590.75^b^	14483.20^a^	469.72^b^	1143.58^b^	15577.25^a^	2336.82	***
39	17799.83^a^	8084.50^b^	6510.91^b^	16969.92^a^	6112.33^b^	2894.94	**
36	6945.83^b^	28631.60^a^	0.00^b^	1352.42^b^	18825.25^a^	4157.68	***
35	22741.58^a^	15955.70^ab^	5177.45^b^	14381.58^b^	21869.25^a^	2304.79	***
34	0.00^b^	9517.70^a^	5154.27^ab^	7973.33^a^	0.00^b^	2354.93	*
32	26401.08^a^	20633.50^ab^	3007.18^c^	6154.33^c^	15488.92^b^	3438.32	***
30	16890.67^a^	7811.10^b^	3576.45^b^	6337.58^b^	3168.67^b^	2135.17	***
26	11176.17^a^	5146.90^ab^	1953.18^b^	6239.42^ab^	0.00^b^	2242.67	*
25.8	14984.58^a^	0.00^c^	3199.63^b^	8670.00^ab^	0.00^c^	3040.27	**
23	25993.75^a^	12776.30^ab^	2580.91^b^	10716.25^ab^	22459.42^a^	5241.73	*
22	8834.25^a^	5983.10^ab^	0.00^b^	2248.33^b^	0.00^b^	2286.75	*
21	15972.08^a^	5985.00^b^	0.00^b^	8908.17^ab^	6336.92^ab^	3048.89	*
20	10370.33^a^	1487.60^b^	1118.18^b^	1436.67^b^	0.00^b^	2373.07	*
17	1265.75b^c^	0.00^c^	0.00^c^	10027.00^ab^	12803.00^a^	3156.96	*
16	2652.92^b^	1451.30^b^	0.00^b^	5038.67^b^	18888.33^a^	4090.57	*
15.3	10095.92^a^	3357.70^b^	3007.27^b^	1251.33^b^	0.00^b^	2048.71	*
14	15648.50^a^	2536.70^bc^	6107.73^bc^	9930.25^ab^	1723.92^c^	2712.30	**
12.8	0.00^b^	0.00^b^	0.00^b^	8061.17^ab^	11825.83^a^	2948.91	*
11.7	13733.67^a^	12939.10^a^	580.09^b^	7786.00^ab^	4109.92^b^	2888.65	*
11	1326.08^b^	2866.10^ab^	8542.09^a^	8257.92^ab^	0.00^b^	2155.24	*
10	1148.67^b^	4624.00^b^	6155.18^b^	19866.17^a^	8096.58^b^	3980.23	*

Per fragment (size kDa) the average extinction (least square means) per line of hens is shown, with standard error (SE) and P-value.

1 Line: = genotypic high hens (N = 12), Cg = control hens (N = 11), Lg = genotypic low hens (N = 11), Ch = phenotypic high hens (N = 12), Cl = phenotypic low hens (N = 12).

Extinctions within a row with different superscripts^a,b,c^ differ significantly (P<0.05).

* P<0.05,

** P<0.01,

*** P<0.001.

For IgTotal, in total 65 fragments were detected, of which 30 fragments showed a significant line effect (Table 4). Beside the fragments for which significant line differences were found, lines did not differ significantly for the following fragments (kDa): 97-, 82-, 76-, 72-, 68-, 66-, 59-, 54-, 53-, 50-, 48-, 47-, 45-, 42-, 41-, 40-, 37-, 35-, 30-, 29-, 26-, 24.8-, 24-, 21.6-, 19-, 18.3-, 16.5-, 16-, 15.7-, 14-, 13.8-, 13.5-, 12.3-, 11-, and 10.6-. IgTotal shared 44 fragments with IgM, and 42 fragments stained with IgG. Twenty fragments of IgTotal were only stained by IgG but not with IgM, two fragments were stained by both IgM and IgG but not by IgTotal. All IgTotal stained fragments were either stained by IgM or IgG. With respect to line effects, the data of the Western blot profiles of chicken IgTotal staining CLL fragments indicated that some fragments were significantly higher stained in one or more of the five different lines. The genetic high (Hg) line was ‘characterized’ by staining of the fragments (in kDa) 62-, 34-, and 17.7-. The random bred control (Cg) line by staining of a 43 kDa fragment, while the genetic low (Lg) line by staining (in kDa) of 93-, 57-, and 28- fragments. The phenotypic high (Ch) line was characterized by staining of (in kDa) 36-, 23-, 19.4-, 15-, 12.8-, and 11.7- fragments, whereas the phenotypic low (Cl) line by staining (in kDa) of 88-, 74-, 23.5-, 22-, 21-, 20.5-, 15.3-, 13-, 12.8-, and 10- fragments. The genetic low (Lg) line was also characterized by a significantly lower staining of a 39 kDa fragment.

For IgM, in total 51 fragments were detected, of which 23 showed a significant line effect (Table 5). Besides these fragments, 28 fragments were stained by IgM, which did not differ significantly between the lines (in kDa): 82-, 74-, 57-, 37-, 30-, 28-, 26.5-, 26-, 25.4-, 24.8-, 24-, 23-, 21-, 19-, 17-, 16-, 15.3-, 14-, 13.8-, 13.5-, 13-, and 10.6-. The IgM staining pattern revealed 44 bands that were also stained by IgTotal. With respect to line effects, the genetic high (Hg) line was characterized by higher staining of fragments (in kDa) 97-, 93-, 66-, 47-, 43-, 29-, and 11.7-. The random bred (Cg) line by higher staining of fragments (in kDa) 27-, 17.7-, 15-, and 12.3-. The genetic low (Lg) line was characterized by lower staining of a 50 kDa fragment. In the phenotypic high (Ch) line higher staining was found of (in kDa) fragments 66- (alike the Hg line), 60-, 47- (alike the Hg line), 17.5-, and 11-. In the phenotypic low (Cl) line higher staining was found of (in kDa) fragments 76-, 39-, 35-, 32-, 20-, 11 (alike the Ch line) and 10-.

For IgG, in total 69 fragments were detected, of which 31 showed a significant effect (Table 6). Besides these fragments, 38 fragments were stained by IgG, which did not differ significantly between the lines (in kDa): 82-, 80-, 76-, 74-, 72-, 68-, 62-, 57-, 54-, 53-, 50-, 48-, 42-, 41-, 37-, 33-, 31-, 29-, 28-, 27-, 25.4-, 24.8-, 24-, 23.5-, 21.6-, 20.5-, 19.4-, 19-, 18.3-, 17.7-, 16.5-, 15.7-, 15-, 13.8-, 13.5-, 13-, 12.3-, and 10.6-. With respect to line effects, the genetic high (Hg) line was characterized with higher staining of fragments (in kDa) 97-, 66-, 47-, 44-, 39-, 35-, 32-, 30-, 26-, 25.8-, 23-, 22- 21-, 20-, 15.3-, 14-, and 11.7-. The random bred (Cg) line showed higher staining of fragments (in kDa) 93-, 44-, 40-, 36-, 34-, and 32-. The genetic low (Lg) line was ‘characterized’ by no staining of (in kDa) 66-, 22-, and 21- fragments. The phenotypic high (Ch) line showed higher staining of fragments (in kDa) 61-, 43-, and 10-, and showed similar staining as the genetic H line for 47-, 43-, 39-, 34-, 26-, 25.8- 23-, 21- 14-. The phenotypic low (Cl) line was characterized by staining of fragments (in kDa) 88-, 43-, 40-, 36-, 17-, 16-, and 12.8-, and shared staining characteristics with the genetic H line (Hg) for the 97-, 66-, and 35- fragments. There were no similarities between the genetic low (Lg) line and the phenotypic low (Cl) line.

Taken together, depending of antibody isotype, every chicken line had different staining intensities to fragments of CLL. Only the genetic low (Lg) line was characterized by a complete lack of staining of certain fragments, but on the whole this line showed staining of most fragments albeit sometimes with lower intensity. Surprisingly, there was much difference in profiles between the genetic high (Hg) line, selected for specific antibody responsiveness to SRBC, and the phenotypic high (Ch) line selected for high natural antibody levels to KLH. On the contrary, there was similarity between the genetic high (Hg) line and the phenotypic low (Cl) line selected for low NAb levels. In this respect, the similarity between the genetic low (Lg) line and the phenotypic low (Cl) line was low. Also, both Ch and Cl lines were different from the random bred Cg line.

### Correlations and clustering of lines by principal component analysis

**Figure 3:**
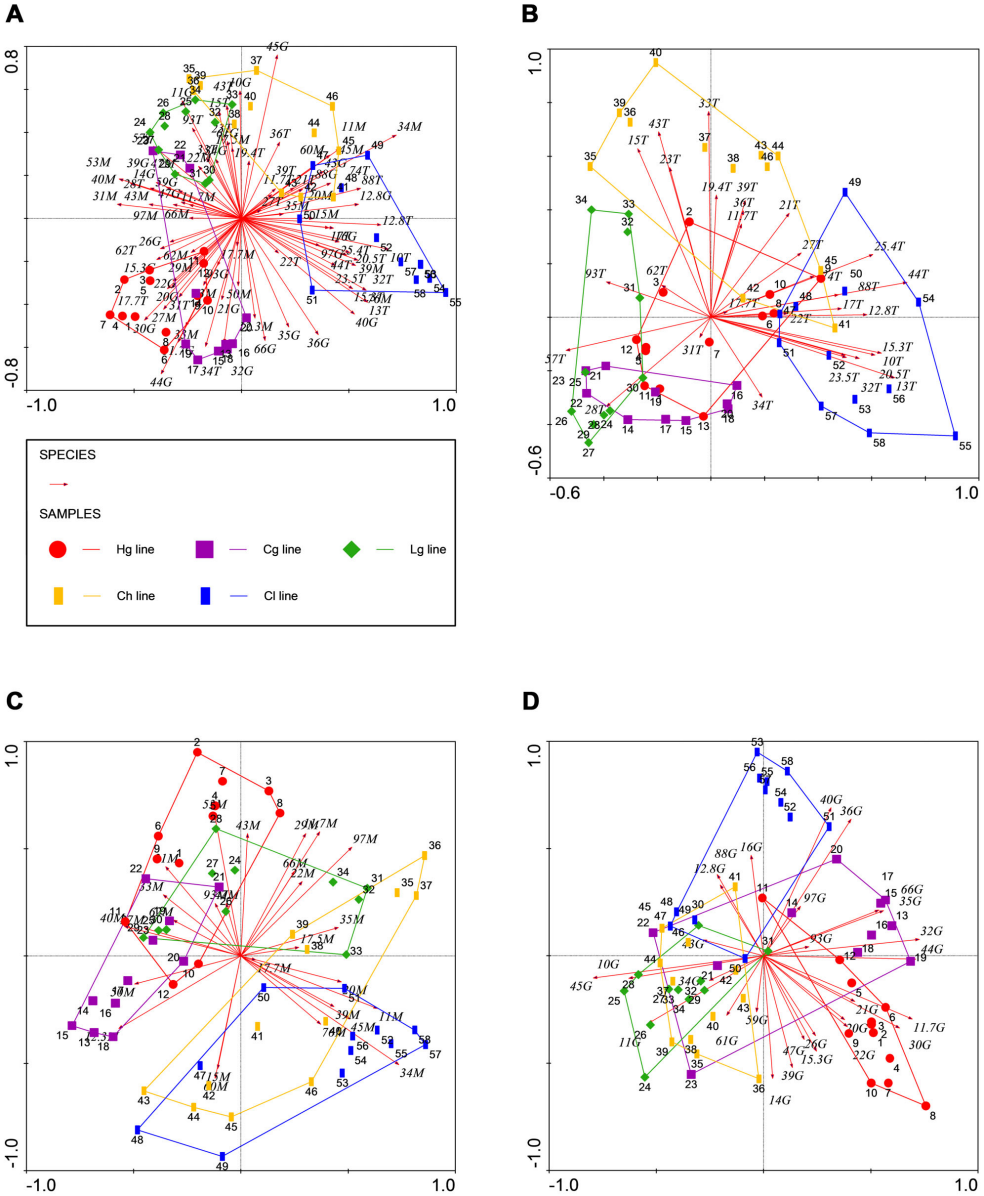
Correlation of staining patterns of all bands stained by all isotypes (A) or stained by IgTotal (B), IgM (C), and IgG (D) autoantibodies to CLL. Chickens were one year of age and divergently selected for 29 generations for High (red circles: Hg), Low (green diamonds: Lg), and Control (purple squares: Cg) agglutination titres to SRBC at 5 weeks of age, and C line birds phenotypically selected for high (yellow bars: Ch) or low (blue bars: Cl) NAb levels to KLH at 16 weeks of age. Ordination plots by PCA. Graphs include 11–12 birds from each line as also used for Figures 1 and 2. Only CLL fragments that showed significant line differences for staining intensity (Tables 4–6) are shown. ‘Eigenwaardes’ were 12.8% horizontal and 11.1% vertical axes (A), 17.9% horizontal and 11.6% vertical axes (B),16.5% horizontal and 15.4% vertical axis (C), and 20.8% horizontal and 12.5% verical axis (D), respectively. Numbers in *Ithalic* represent CLL fragments most informative for line clustering.

Binding of all three antibody isotypes (IgTotal, IgM, and IgG) in plasma of one year old chickens to CLL revealed clustering of the binding patterns of the five lines for all three separate isotypes (Fig. 3). Fig. 3A shows clustering of the lines on the basis of all fragments of CLL stained by all isotypes (IgTotal, IgM and IgG) and which were significant as shown in Tables 4, 5, and 6. On first glance there were many correlations between bands of different molecular weight and stained by different isotypes which contributed to the clustering of the five lines. The Hg line and the Lg line revealed a condense clustering suggesting homogeneity of these lines in their auto-antibody profiles, whereas the Cg, Ch and Cl lines showed larger areas of clustering suggesting more heterogeneity in their auto-antibody profiles. The Cg line overlapped both the Hg and the Lg line, whereas the Ch and Cl line were separately clustered (Fig. 3A). Clustering was also apparent when the isotypes were separately analysed in PCA (Figs. 3B, C, and D). In Fig. 3B the clustering of the lines on the basis of their auto-IgTotal is shown. Five different clusters were found with relatively little overlap. The Cg line overlapped the Hg and the Lg line, but there was no overlap with the Ch and Cl lines. Similar was true for the IgM isotype: the Cg cluster overlapped the Hg and the Lg clusters, but there was no overlap with the Ch and Cl clusters. In Fig. 3D it is shown that again the Hg and the Lg line show different clusters on the basis of the auto-IgG profiles, which are overlapped by the Cg line. The Ch line is related to the Lg line, whereas the Cl line represents a different separate cluster. Taken together, the Hg line could be characterized by the following bands: 62T, 34T, 31T, 17.7T, 97M, 93M, 43M, 29M, 11.7M, 30G, 22G, 21G, and 20G, the Lg line was characterized by 57T, 27T, and 11G. The Cg line by 50M, 12.3M, and 93G, the Ch line by 44T, 33T, 21T, 15T, 11.7T, 17.5M, and 61G, and finally the Cl line by 23.5T, 20.5T, 15.3T, 13T, 12.8T, 76M, 39M, 35M, 34M, 40G, 36G, and 16G fragments. The Cl line was completely different from the Lg and the Cg lines, whereas also the Ch line was completely different from the Hg and the Cg lines. A considerable fraction of the Ch line was clustered alike the Lg line.

## Discussion

In a previous study [19], we described the presence of antigen-binding antibodies in healthy non-immunized chickens from lines selected for high or low specific antibody responses to SRBC. Different and high levels of these so-called natural antibodies (NAb) were found in the three lines. The levels of natural antibodies binding various antigens as determined by ELISA differed between the three selection lines. In general, chickens genetically selected for high specific antibody responses to SRBC (Hg line) also showed higher levels of NAb binding various antigens [19]. In the same study, we also found, but did not quantitate, plasma antibodies binding extracts of chicken heart, spleen, liver, brain, bursa, thymus and kidney, that may reflect so called natural auto-antibodies (N(A)Ab). In the present study, we found different levels of N(A)Ab binding CLL in the three lines, with highest levels, as expected in the Hg line (Table 1). However, ELISA approaches do not reveal the specificity of the antigenic fragments present in a complex antigen mixture such as CLL. Therefore, we performed Western blot analysis to further study the nature of the antigens in CLL bound by auto-antibodies, and to establish whether the difference between the lines reflected levels of antibodies binding auto-antigens or also included differences in antibody idiotypes between the three selection lines. In addition we included in the present study two lines that originated from the Cg line, and which were phenotypically selected for high or low NAb levels binding KLH (Ch and Cl lines).

The present study revealed that plasma of one year old healthy chickens contained auto-antibodies of both the IgM and the IgG isotype to large numbers of chicken liver lysate fragments (CLL). Within individual birds, the fragments bound by IgM were not always similar as the fragments bound by IgG, and within isotypes (IgTotal, IgM and IgG), there was a large variety of fragments stained by individual birds. However, fragments were found that were significantly stained more intense in one or more of the lines, or stained much less or not at all in one or more of the lines. Also, PCA revealed distinct clustering of the five lines regardless of isotype. These results suggested 1. that birds can be individually typed by an auto-antibody fingerprint, and 2. genetically or phenotypically related birds are clustered together based on their auto-antibody fingerprints. The latter implies that (groups of) individuals grouped outside a fingerprint cluster might reveal different genotypes or phenotypic traits. This may provide a tool to relate auto-antibody profiles with genetic background (current Hg and Lg lines), and phenotypic traits such as immune responsiveness (current Ch and Cl lines), immune modulation, disease resistance and (metabolic) disorders.

In the present study we used two lines divergently selected during 29 generations for either high (Hg line) or low (Lg line) specific antibody titres to SRBC at day 5 after immunization at 5 weeks of age, next to a random bred control (Cg) line, that resembles the original parental stock, and two lines consisting of C line birds that were phenotypically selected at 16 weeks of age for high (Ch line) or low (Cl line) natural antibody (NAb) ‘titres’ to KLH. The Hg line is characterized by higher levels of T-cell dependent specific antibodies, but also has more NAb to almost all ‘antigens’ as compared to the Cg and Lg lines [19]. The Lg line is characterized by low T-cell dependent antibody responses. Thus using these birds we studied both genotypic as well as phenotypic contrasts. In addition, two lines (Ch and Cl) were phenotypically selected for NAb levels binding KLH. Also these two phenotypically distinct lines showed distinct clustering of auto-antibodies to CLL.

The present study revealed that IgTotal as well as the isotypes IgM and IgG in plasma from individual chickens stained many fragments of chicken liver. The highest number of distinct bands for the isotype IgG as found in the present study is in line with results found for IgG autoantibodies detected in man [14], [23]. In healthy humans, IgM and IgG autoantibody staining patterns were reported to be remarkably similar, i.e. IgG patterns were comprised of the same fragments as IgM, with the addition of few other IgG-specific fragments [23]. On the whole, this is also true for the present results with chicken autoantibodies, most CLL fragments bound by IgM were also bound by IgG. Only two bands bound by IgTotal were neither bound by IgM or IgG. Significant differences of fragments stained by either IgM or IgG differed, however, between the lines. Fig. 3A revealed that clustering of the separate lines was based on binding of the three isotypes to seemingly unrelated fragments. This implies that IgM or IgG (or IgTotal) binding profiles may reveal different forms of information with respect to genotypes and phenotypes. Fig. 2 illustrated the generally observed differences in specificity between the different isotypes binding CLL, when regarding both the background staining as well as specifically distinctly stained fragments. In this figure, three graphs from the same individual show increased background staining in the order of IgG, IgM and IgTotal. The height of the specific peaks, however, decreases in the same order. These results suggest that IgG might be the most specific immunoglobulin having the least background staining and the highest specific peaks. This might be a consequence of the prototypical affinity maturation of IgG-producing B-cells, increasing the average affinity of antigen-specific antibodies but additionally also increasing the intensity of band staining of cross-binding auto-antigens. In addition, between individuals, IgG staining of CLL was much more variable as compared to IgM or IgTotal. This reaffirms the suggestion that auto-IgG staining profiles might be the most suitable for antibody ‘fingerprinting’ [17]. Also Mouthon et al. [23] showed significant inter-individual differences in the intensity and nature of immune reactivity for IgG in man, and that amongst other tissues, the highest degree of heterogeneity and stability of IgG reactivity was found for liver. This suggested that also in the current study with chickens, liver antigens were appropriate to study the auto-antibody repertoire. Especially IgG staining profiles were very different between individuals. The chicken lines used in the current study were not inbred. In the Hg line, there were three full sibs (3, 2, and 2 sisters, respectively, out of 12 birds), in the Cg line 1 full sib (2 hens out of 11), in the Lg line 1 full sib (2 sisters out of 11), and in the Cl line also 1 full sib (2 sisters out of 12). The number of full sibs was too low to analyse family affects.

Western blotting with biological material likely illustrates biological relevant antibody reactivity, but repeatability of these assays may be not as high as when using proteomic approaches, and therefore interpretations require caution. Repeatability of specific molecular band weights in Western blotting is low (generally varying from 60–80%) but patterns are repeatable between separate blots. Fig. 3A shows that clustering of the lines based on all binding profiles albeit this figure rests on the use of 15 different blots. This indicated that not individual specifically stained bands, but profiles are probably more useful to type and cluster the chicken lines or individual chickens. Western blotting under reducing conditions as used in the present study detects mainly linear epitopes. It is likely that when using different gels or conjugate dilutions, different electrophoresis conditions or different procedures for CLL preparation, results may be different. Also, a relatively limited number of individuals (11–12) were studied per line and at one specific age. However, we repeated the current Western blot analysis with 15 other individuals of the same age of the Hg, Cg, and Lg lines, respectively, and found similar separate clustering of the lines as in the current study (data not shown). Whether the profiles are stable or change in time (aging), or after immune challenge within individual chickens is currently under study. As yet we have no evidence that allo-antigens were stained in the current study.

Table 3 indicated that hens genetically selected for lower specific antibody responses (Lg line) on average showed the lowest number of CLL fragments recognized by auto-antibodies, and thus may be characterized by less extensive N(A)Ab profiles. On the other hand, C line birds phenotypically selected for low NAb levels to KLH, the Cl birds, did not show a remarkably lower number of fragments recognized nor significantly lower intensity staining of the fragments. This suggests a. that genetic selection for 29 generations for specific antibody responses at a young age cannot be compared with phenotypic selection for natural antibody levels at an older age, or that selection for NAb levels to KLH in one generation has no relation yet with the repertoire of autoantibodies binding CLL. The clustering in Fig. 3 showed that for IgG and IgTotal, genetic selection on lower specific, but also genetic selection for higher specific antibody responses (to SRBC) resulted in a lower number of stained CLL fragments indicative for these lines as compared to the Cl and Ch lines suggesting a restriction in auto-antibody repertoire due to divergent selection (Hg and Lg). It was surprising that irrespective of the isotype, the Cg, Cl and Ch lines were distinct clusters, indicating a flexibility in the random bred C line population which is not found any more in the Hg and Lg selection lines. This would argue for an advantage in the C lines for establishing a dynamic diversity of the antibody repertoire contributing to molecular recognition by facilitating the induction of an immune response against the broadest range of foreign molecules. Alternatively, birds of the Hg and Lg selection lines show a restricted antibody repertoire providing an advantage for some antigens but nor for many others [2]. Regardless of genetic or phenotypic selection, either selection on specific or natural antibodies, significant line effects were found in the binding of the antibodies to CLL, which was strengthened by the distinct PCA clustering of the five lines for their binding pattern to CLL. The differences in profiles of different genetic (and phenotypic) lines found in the present study with chickens were in line with the (inbred) strain differences found in mice [18], which was related with the C5 component of complement. This implied that minor genetic differences already could have a visible effect on autoantibody profiles.

We expect that the extensive staining patterns in the present study were influenced by the age of the birds studied. During aging different components of the immune system undergo different changes [24], like a reduction in antibody specificity, affinity, and isotype switch [25], [26], [27], which all increase susceptibility to disease. Lacroix-Desmazes et al. [12] found decreasing auto-IgG reactivity with age. NAb levels in poultry increased with age [19], [28]. This implies that changes occur in different directions, both increasing and reducing different mechanisms. In humans, increasing age was associated with increased idiopathic paraproteinaemia [29] and changes in the antibody repertoire [27], which might underlie the increase in the binding of autoimmune antigens. In man, the initial little organisation of the natural autoantibody repertoire at birth results in a modular organization of subgroups of correlated (auto-) and exo-antigens in young adults [30], [31]. Babies are born with a universal IgM repertoire next to a maternal ‘inherited’ IgG repertoire that integrates during aging. These results strongly suggest a continuous development or maturation of the N(A)Ab production during life. Whether similar is true for chickens awaits further studies. For mice it was shown that the repertoire of serum (auto-)IgM was largely independent of external antigenic contact [32], whereas levels were affected by the hygienic status of the animal.

In poultry, higher NAb titres to the complex antigen KLH were related with higher survival rates in poultry [5], [33]. Higher levels of anti-nuclear antibodies in sheep correlated with higher survival [34]. In the present study per chicken on average 14–20 fragments and per line approximately 60 fragments were stained. Quintana and Cohen [35] concluded by studying murine strains that the repertoire (network) of N(A)Ab reflects susceptibility to develop autoimmune disease. Also healthy humans and individuals with type 1 or type 2 diabetes mellitus, or Behçet's disease patients were distinguished by means of clusters of N(A)Ab profiles. It is tempting to speculate that also in poultry, differences in fingerprints or binding profiles of N(A)Ab could function as an indicator of health, disease or disorder. The quantity, but not the idiotype, of IgM auto-antibodies in mice was related with the hygienic status of the diet suggesting an important role of the microbiota in the formation of N(A)Ab [32]. In man, IgG autoantibody profiles were reported to be unique to an individual, and albeit stable in time, influenced by gender, age and the presence of diseases [36]. We propose that quantitative idiotype mapping of N(A)Ab isotypes, i.e. idiotype fingerprints from either IgM or IgG, or both provide a future tool for diagnosis or prediction of diseases [37], effects of immunomodulation, or estimation of effects of breeding or management procedures for poultry.
